# Integrated Analysis of Stemness-Related LncRNAs Helps Predict the Immunotherapy Responsiveness of Gastric Cancer Patients

**DOI:** 10.3389/fcell.2021.739509

**Published:** 2021-09-13

**Authors:** Quan Jiang, Lingli Chen, Hao Chen, Zhaoqing Tang, Fenglin Liu, Yihong Sun

**Affiliations:** ^1^Department of General Surgery, Zhongshan Hospital, Fudan University, Shanghai, China; ^2^Human Phenome Institute, Fudan University, Shanghai, China; ^3^Department of Pathology, Zhongshan Hospital, Fudan University, Shanghai, China

**Keywords:** gastric cancer, stemness, lncRNA, prognosis, immunotherapy

## Abstract

The immune microenvironment plays a critical role in tumor biology. As a critical feature of cancers, stemness is acknowledged as a contributor to the development of drug resistance in gastric cancers (GCs). Long non-coding RNAs (lncRNAs) have been revealed to participate in this process. In this study, we aimed to develop a stemness-related lncRNA signature (SRLncSig) with guiding significance for immunotherapy. Three cohorts (TCGA, Zhongshan, and IMvigor210) were enrolled for analysis. A list of stemness-related lncRNAs (SRlncRNAs) was collected by co-expression strategy under the threshold of coefficient value >0.35 and *p*-value < 0.05. Cox and Lasso regression analysis was further applied to find out the SRlncRNAs with prognosis-predictive value to establish the SRLncSig in the TCGA cohort. IPS and TIDE algorithms were further applied to predict the efficacy of SRLncSig in TCGA and Zhongshan cohorts. IMvigor210 was composed of patients with clinical outcomes of immunotherapy. The results indicated that SRLncSig not only was confirmed as an independent risk factor for GCs but also identified as a robust indicator for immunotherapy. The patient with a lower SRLncSig score was more likely to benefit from immunotherapy, and the results were highly consistent in three cohorts. In conclusion, our study not only could clarify the correlations between stemness and immunotherapy in GC patients but also provided a model to guide the applications of immunotherapy in clinical practice.

## Introduction

As a heterogeneous disease with a worldwide presence, gastric cancers (GCs) are ranked as the fifth most common malignant tumor and the fourth leading cause of cancer-related mortality worldwide (Bray et al., 2018; Sung et al., 2021). Despite all the efforts devoted to improving the curative effect in the last decade, the treatment outcomes of GC remain unsatisfactory. As is concluded in previous studies, more than two-thirds of GC cases are diagnosed at advanced stages globally (In et al., 2017). Compared with the treatment outcomes in Western countries, GC patients in eastern Asia are diagnosed at a relatively early stage and thus have better survival. However, there is still much room for improvement in treatment outcomes since the median overall survival is only 11 months with combination chemotherapy (Jin et al., 2020). Later lines of systemic therapy, such as ramucirumab and trifluridine/tipiracil, improve survival by only 1-2 months compared with placebo. New effective therapeutics are needed (Fuchs et al., 2014; Shitara et al., 2018).

Immune checkpoint blockade (ICB) has brought about unprecedented hope for cancer patients losing the opportunity for radical surgery, yet the clinical benefits are still limited. For the ICB treatments of GCs, the current circumstances are not satisfactory. In 2017, a programmed cell death-1 (PD-1) inhibitor named pembrolizumab was granted accelerated approval from the United States Food and Drug Administration for the treatment of recurrent locally advanced or metastatic GCs. Approval was made based on a single-armed phase 2 clinical trial (Fashoyin-Aje et al., 2019). Previous studies hold the opinion that tumors with higher expression levels of programmed cell death ligand 1 (PD-L1) are more likely to benefit from PD-1 inhibitors. However, the objective response rate in PD-L1-high tumors was finally revealed to be 11.6 and 11.2% in two independent PD-1 inhibitor-concerning clinical trials (Kang et al., 2017; Fuchs et al., 2018). The development of a model for the proper selection of GC patients who can benefit from ICB therapy is urgently needed.

Stemness has long been recognized as one of the most important characteristics of tumor cells. It is acknowledged that stemness may lead to tumor recurrence and chemotherapy resistance. However, it remains unknown whether stemness may affect the efficacy of immunotherapy in GCs. Defined as transcripts that are longer than 200 nt, long non-coding RNAs (lncRNAs) have been identified as crucial factors affecting tumor biological function. The aberrant expression of lncRNAs has been revealed to have a profound impact on cancer biological behaviors such as proliferation, progression, and metastasis (Sanchez Calle et al., 2018). The correlations between lncRNAs and cancer stem cells (CSCs) have been revealed in previous studies (Sanchez Calle et al., 2018). Many lncRNAs have been reported to affect the biological functions of cancer by regulating the stemness of cancer cells. It has been reported that DANCR could enhance the stemness characteristics of hepatocellular carcinoma by lowering the expression of CTNNB1, which thus leads to chemotherapy resistance (Yuan et al., 2016). Moreover, the development of CSCs in breast cancers was found to be highly linked with a novel lncRNA named lnc030, which may stabilize SQLE mRNA and increase stemness features (Qin et al., 2021). Furthermore, stemness-related genes (SRGs) have also been reported to be correlated with tumor immunity. Hao et al. conducted a network analysis to collect SRGs in lung cancers. The genes were further processed to establish a signature with predictive value for chemotherapy and immunotherapy responses (Zeng et al., 2020b). Recent evidences have also suggested that lncRNAs contribute to the malignant phenotypes of cancer not only through genomic or transcriptomic alterations but also by altering the immune microenvironment (Atianand et al., 2017).

In this study, we aimed to develop a stemness-related lncRNA signature (SRLncSig) with guiding significance for immunotherapy. This study not only provided a novel signature with prognostic predictive value in GC patients but also established a model to predict GC patients’ sensitivity to immunotherapy.

## Materials and Methods

### Data Source

The RNA-seq data and clinical profiles of GC patients were derived from the TCGA Data Portal (December 10, 2020)^1^. A total of 375 patients who had integral lncRNA and mRNA expression profiles, survival information, and common clinicopathological characteristics were enrolled in this signature establishment process. The genomic mutation data (including somatic mutation and copy number variation) of TCGA-STAD were collected from the TCGA Data Portal (see text footnote 1, December 10, 2020). RNA-seq and survival data of 29 GC patients from Zhongshan Hospital enrolled in a clinical trial concerning neoadjuvant chemotherapy were also acquired. The R package “RCircos” was employed to plot the copy number variation landscape of 42 SRGs in human chromosomes. Mandard tumor regression grade (TRG) was applied as the criteria for calculating the chemotherapy response of patients (Mandard et al., 1994). This system classifies pathologic response as follows: TRG 1 (complete regression/fibrosis with no evidence of tumor cells), TRG 2 (fibrosis with scattered tumor cells), TRG 3 (fibrosis and tumor cells with a dominance of fibrosis), TRG 4 (fibrosis and tumor cells with a dominance of tumor cells), and TRG 5 (tumor without evidence of regression). Grade 1-2 was defined as major response while Grade 3-5 was defined as minor response. The transcriptome data and clinical characteristics of the IMvigor210 cohort composed of patients receiving PD-L1 blockade immunotherapy were acquired from the “IMvigor210CoreBiologies” package and normalized by “limma” and “deseq” packages in R software.

### Collection of Stemness-Related LncRNAs

GTF files were downloaded from Ensembl^2^ for annotation to distinguish the mRNAs and lncRNAs. A list of SRGs was acquired from previously published literature (Ramos et al., 2017; Zhao et al., 2017; Saygin et al., 2019; Supplementary Table 1) and was used to screen stemness-related lncRNAs (SRlncRNAs) by a co-expression strategy under the threshold of coefficient value >0.35 and *p*-value < 0.05. Univariate Cox regression analysis was further applied to find out the SRlncRNAs with prognosis-predictive value.

### Construction and Validation of SRLncSig

The integral TCGA samples were randomly assigned into two groups, namely, training cohort (*n* = 187) and testing cohort (*n* = 184). Lasso regression was performed with 10-fold cross-validation and a *p*-value of 0.05 based on the results of the univariate analysis for SRlncRNAs. A total of 1,000 cycles were run, and random stimulation was performed 1,000 times in each cycle. Next, the frequency of each lncRNA was acquired, and lncRNAs with frequencies greater than 100 were collected for further Cox regression analysis. The lncRNAs with independent prognostic predictive value were finally enrolled for the construction of the model with the following formula:


$$SRLncSigscore=\sum_{i=1}^{n}coef{\left(lncRNA\right)}^{*}expr\left(lncRNA\right)$$

In this formula, expr (lncRNA) indicated the expression of lncRNA. Coef (lncRNA) was the predictive value of lncRNA for SRLncSig-based risk scores, which were obtained from the multivariate Cox analysis. Patients in the training cohort were divided into high- and low-risk groups based on risk scores.

### Functional Enrichment Analysis

Using the median value as the cutoff value, two risk stratifications were divided to conduct encyclopedia of Genes and Genomes (KEGG) analysis based on the “cp.kegg.v7.4.symbols.gmt” reference package in GSEA software.

### Tumor Mutation Burdens

Non-synonymous mutation (including frameshift mutation, inflame mutation, missense mutation, non-sense mutation, and splice site mutation) counts were recognized as tumor mutation burdens (TMBs). The TMB score was acquired by calculating the mutation frequency with the number of variants/the length of exons (38 million) for each sample *via* Perl scripts based on the JAVA8 platform.

### Evaluation of the Chemotherapy and Immunotherapy Response Based on SRlncRNA Signature

To explore the sensitivity of each GC patient from TCGA to different chemotherapeutic agents, the “pRRophetic” package in R software (3.6.1) was applied to predict the IC50 of GC-related chemotherapy drugs. The high- and low-risk groups were compared. This algorithm was previously published and had been widely used in multiple studies (Geeleher et al., 2014; Zeng et al., 2020a; Lu et al., 2021; Wang et al., 2021). As is mentioned above, the patients included in the Zhongshan cohort had all received neoadjuvant chemotherapy. The pathological results were acquired to compare the response variation between two risk stratifications. Immunophenoscore (IPS) of the TCGA STAD project was downloaded from The Cancer Immunome Atlas (TCIA)^3^ (Supplementary Table 2). PD1 and CTLA4 were the candidate immune checkpoints enrolled for IPS analysis. Higher IPS represented better accuracy for the more corresponding result. The online tool Tumor Immune Dysfunction and Exclusion (TIDE)^4^ was used to predict the immunotherapeutic responses of each sample of TCGA and Zhongshan cohorts based on the transcriptome profiles. PD1 and CTLA4 were also the candidate immune checkpoints and a lower TIDE score indicated a relatively better response to immunotherapy. The response results of immunotherapy for the IMvigor210 cohort were available in the “IMvigor210CoreBiologies” package in R software. The SRLncRNA was calculated based on the expression level and correlation coefficient of six SRlncRNAs (LINC01094, ADAMTS9-AS1, LINC01614, LINC00449, RNF144A-AS1, and MAPKAPK5-AS1) included in the signature since the rest of the lncRNAs were not available in the IMvigor210 cohort.

### Statistical Analyses

The statistical analyses in this study were all generated by R-3.6.1. For quantitative data, statistical significance for normally distributed variables was estimated by Student’s *t*-tests, and non-normally distributed variables were analyzed by the Wilcoxon rank-sum test. For comparisons of more than two groups, Kruskal-Wallis tests and one-way analysis of variance were used as non-parametric and parametric methods, respectively. Spearman method was applied for correlation analysis between two continuous variables. Two-sided Fisher exact tests were used to analyze contingency tables. Kaplan-Meier survival analysis and the Cox proportional hazards model were used to analyze the association between the two risk stratifications with the R package “Survminer.” The receiver operating characteristic (ROC) curve was used to assess the prognosis classification performance of the SRLncSig score model, and the area under the curve (AUC) was calculated using the “timeROC” package.

## Results

### The Landscape of Genetic Alterations of SRGs in GC

**FIGURE 1:**
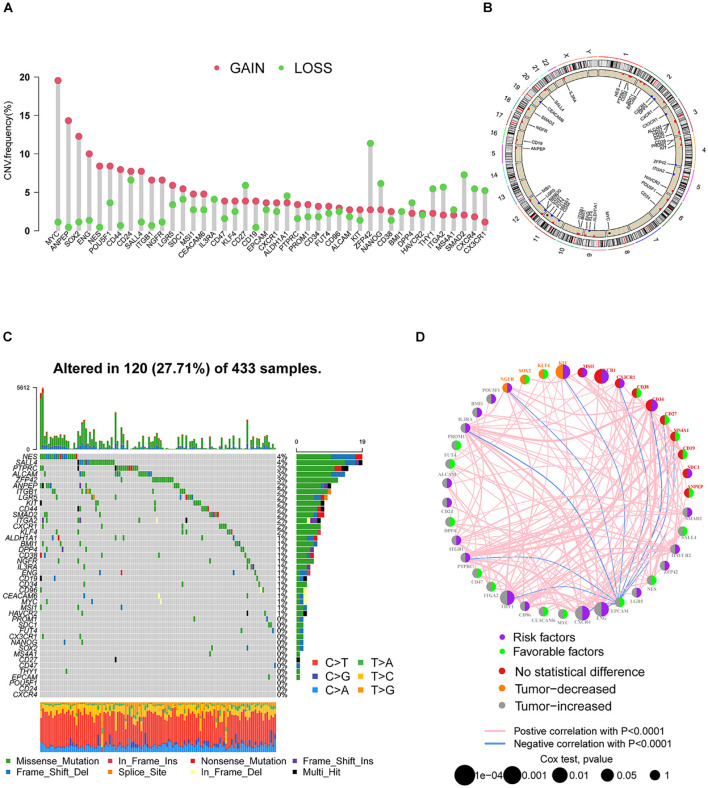
The landscape of genetic alterations of SRGs in GC. **(A)** The CNV mutation frequency of SRGs was prevalent. The column represented the alteration frequency. The deletion frequency, green dot; the amplification frequency, red dot. **(B)** The locations of CNV alteration of SRGs on chromosomes. **(C)** Somatic mutation spectrums of SRGs were presented by a waterfall map. **(D)** The interaction of expression on SRGs in GC. The expression variations of SRGs between normal and tumor tissues were depicted by circles in different colors in the left half of the circle. No statistical difference, red; tumor decreased, orange; tumor increased, gray. The lines connecting SRGs represented their interaction with each other. The size of each circle represented the prognosis effect of each regulator and scaled by *p*-value. Favorable factors for patients’ survival were indicated by a green dot in the right half of the circle and risk factors indicated by the purple dot in the right half of the circle.

A total of 42 SRGs were included in this study (Supplementary Table 1). To determine the genetic alterations in SRGs in GC, the prevalence of copy number variations (CNVs) and somatic mutation variations (SNVs) was assessed. The CNV mutation frequency of SRGs is presented in Figure 1A. The rates of amplification and deletion for SRGs were both low and the locations of CNV alterations of 42 SRGs on chromosomes are also shown (Figure 1B). Out of 433 samples, 120 were detected harboring mutations of SRGs (Figure 1C). Though the mutation frequency of individual SRGs was relatively low, most of the genes were revealed to harbor mutations in GC. The comprehensive landscape of the interactions between 42 SRGs in GC patients was illustrated in the network (Figure 1D). Most of the SRGs increased in tumor tissues and more than half of SRGs indicated worse prognosis (Figure 1D). Most of the SRGs were positively interconnected (Figure 1D). The results indicated that the cross-talk among SRGs probably played critical roles in the formation of GCs and was implicated in cancer progression.

### Identification of SRlncRNAs in GC

**FIGURE 2:**
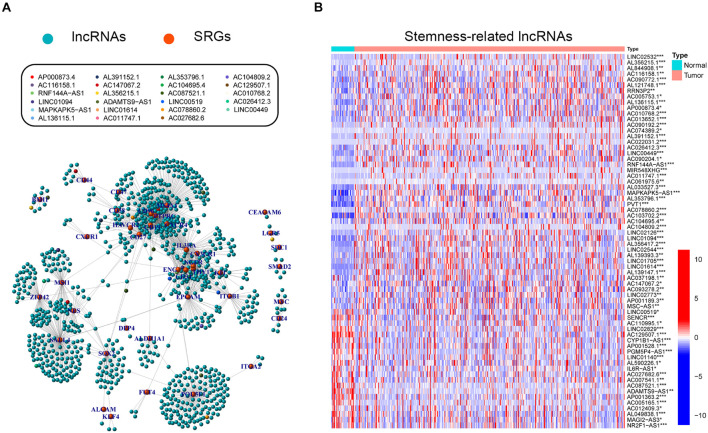
Exploration of stemness-related lncRNAs in GC. **(A)** Correlative network of SRLncRNAs and SRGs; blue balls represented lncRNAs while orange ones represented mRNAs. Twenty-three lncRNAs enrolled to establish the following signature were marked with different colors. **(B)** Comparisons of SRLncRNAs with prognosis-predictive value between tumor and normal tissues (Kruskal-Wallis test, *p < 0.05; **p < 0.01; ***p < 0.001).

Stemness-related lncRNAs were identified based on co-expression analysis of SRGs and lncRNAs in the TCGA cohort. A total of 929 lncRNAs were identified as SRlncRNAs (Figure 2A and Supplementary Table 3). After that, univariate Cox regression analysis was performed based on the SRlncRNAs, and 66 out of 929 SRlncRNAs were revealed as prognosis-associated. It is interesting that all the 66 lncRNAs were differentially expressed between normal and tumor tissues (Figure 2B). Afterward, the 66 SRlncRNAs with prognostic value were extracted to conduct Lasso regression analysis (Supplementary Figure 1 and Supplementary Table 3). Twenty-three SRlncRNAs were finally collected for signature establishment (Figure 2A and Supplementary Table 3). The correlation analysis among the 66 lncRNAs also indicated that most of them were positively linked (Supplementary Figure 2). Therefore, the interaction and biological function of the SRlncRNAs are worthy of further studies.

### Establishment and Validation of the Robustness of SRLncSig

**FIGURE 3:**
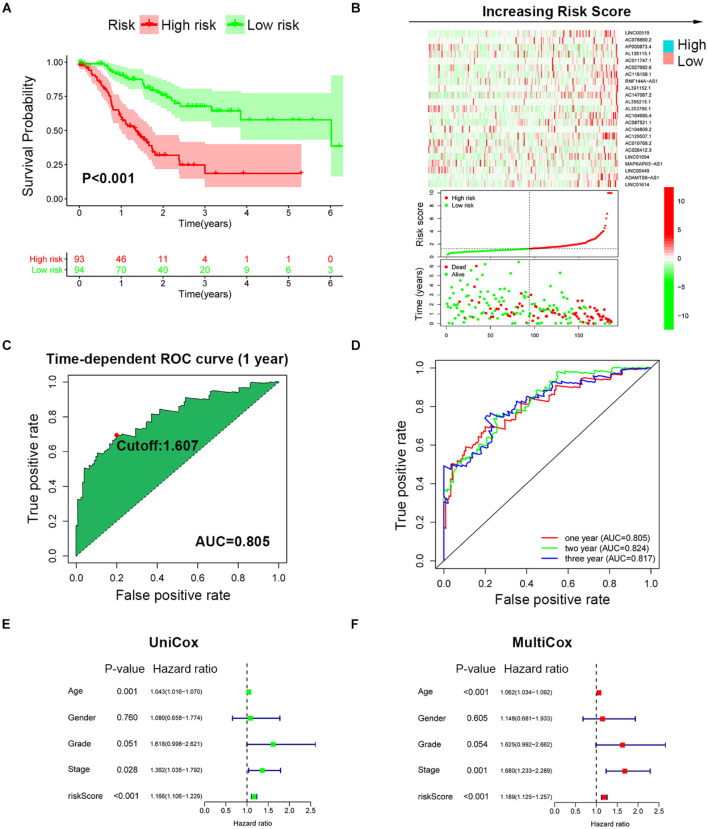
Establishment of SRLncSig based on the training cohort. **(A)** Kaplan-Meier estimates of overall survival of patients with low or high risk predicted by the SRLncSig revealed that the low-risk group presented with a better survival. **(B)** The signature separated the integral cohort into low- and high-risk groups based on the medians of the risk score (horizontal lines: median values). **(C)** The time-dependent ROC curve presented with an AUC value of 0.805 for the signature and 1.607 was set as the cutoff to separate the two risk stratifications. **(D)** The AUC values of the 1-, 2-, and 3-year ROC curve were 0.805, 0.824, and 0.817, respectively. **(E,F)** Univariate and multivariate Cox regression analysis confirmed SRLncSig as an independent prognostic factor for the training cohort.

To improve the prognostic predictive value of the SRlncRNAs, patients of the TCGA cohort were randomly divided into two cohorts, namely, a training cohort (*n* = 187) and a testing cohort (*n* = 184). Twenty-three SRlncRNAs acquired from Lasso regression analysis were extracted to calculate the risk score based on the formula mentioned above (Supplementary Figure 3). In the training cohort, a Kaplan-Meier curve was applied, and it was found that the low-risk group had better survival compared to the high-risk group (*p* < 0.001) (Figure 3A). The signature distinguished the integral cohort into low- and high-risk groups based on the median of the risk score (Figure 3B). The time-dependent ROC curve presented with an AUC value of 0.805 for the signature and 1.607 was set as the cutoff to separate two risk stratifications (Figure 3C). The AUC values of the 1-, 2-, and 3-year ROC curve were 0.805, 0.824, and 0.817, respectively (Figure 3D). The univariate and multivariate Cox regression analyses finally confirmed the signature as an independent risk factor for GCs [*p* < 0.001, HR = 1.189, 95% CI (1.125–1.257)] (Figures 3E,F). Consistently, TNM stage [*p* = 0.001, HR = 1.680, 95% CI (1.233–2.289)] was also revealed as an independent risk factor (Figures 3E,F).

**FIGURE 4:**
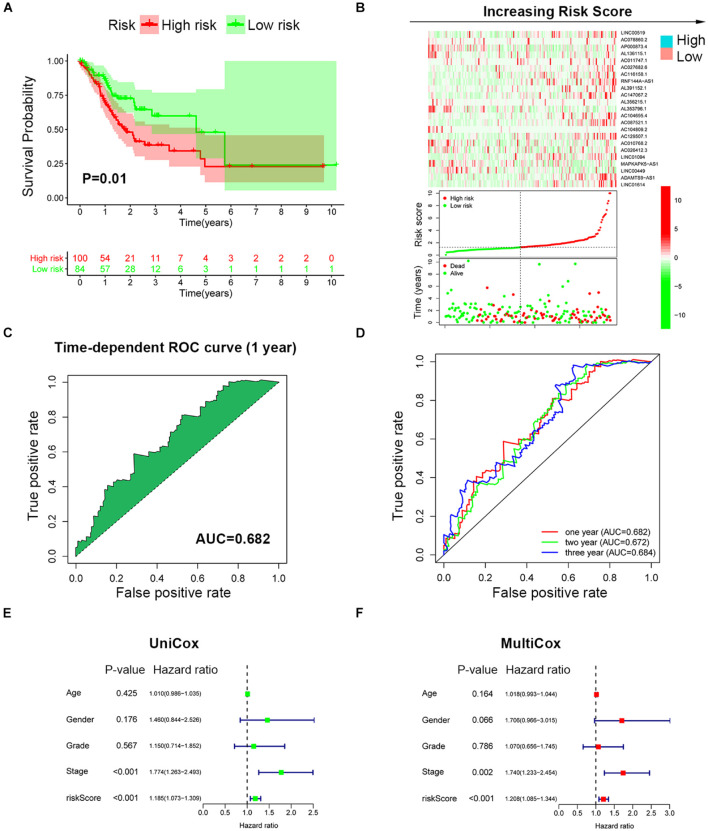
Validation of SRLncSig based on the testing cohort. **(A)** Kaplan-Meier estimates of overall survival of patients with low or high risk predicted by the SRLncSig confirmed that the low-risk group presented with a better survival. **(B)** The signature separated the integral cohort into low- and high-risk groups based on the cutoff value acquired from the training cohort analysis (horizontal lines: 1.607). **(C)** The time-dependent ROC curve presented with an AUC value of 0.682 for the signature. **(D)** The AUC values of the 1-, 2-, and 3-year ROC curve were 0.682, 0.672, and 0.684, respectively. **(E,F)** Univariate and multivariate Cox regression analysis also confirmed SRLncSig as an independent prognostic factor for the testing cohort.

**FIGURE 5:**
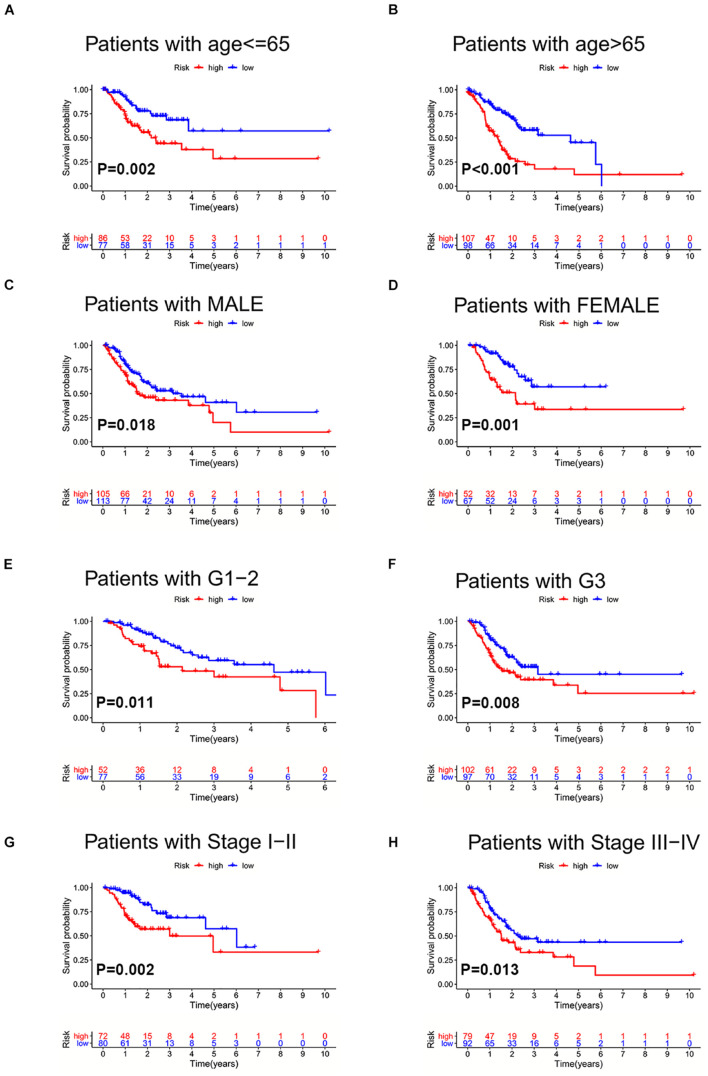
Validation of SRLncSig in different clinical sets. SRLncSig exerted an effective prognosis-predictive value in non-elderly and elderly **(A,B)**, male and female **(C,D)**, lower and higher grade **(E,F)**, and early and advanced stage **(G,H)** for TCGA GC patients.

To further validate the prognosis-predictive efficacy of SRLncSig, the testing cohort was also applied for analysis. All the results were highly consistent with the results of the training cohort. By setting 1.607 as the cutoff risk score value, the low-risk group also presented with better survival than the high-risk group (*p* = 0.01) (Figures 4A,B). The time-dependent ROC curve presented with an AUC value of 0.682 for the signature (Figure 4C) and the AUC values of the 1-, 2-, and 3-year ROC curve were 0.805, 0.824, and 0.817, respectively (Figure 4D), in the testing cohort. The univariate and multivariate Cox regression analyses also confirmed the signature as an independent risk factor for GCs [*p* < 0.001, HR = 1.208, 95% CI (1.085–1.344)] (Figures 4E,F). Consistently, TNM stage [*p* < 0.001, HR = 1.740, 95% CI (1.233–2.454)] was also revealed as an independent risk factor (Figures 4E,F). As for the clinical baseline of the training and testing cohorts, all the characteristics were not significantly variated (Supplementary Table 1). The predictive efficacy of SRlncRNAs was further tested based on different clinical characteristics. The results indicated that SRlncRNAs were still a good prognostic predictor regardless of age, sex, tumor grade, and TNM stage (Figures 5A–H). The results indicated that the accuracy of SRLncSig for prognosis prediction was robust.

### Functional Enrichment Analysis and Interrelations Between the SRLncSig Score and SNVs

**FIGURE 6:**
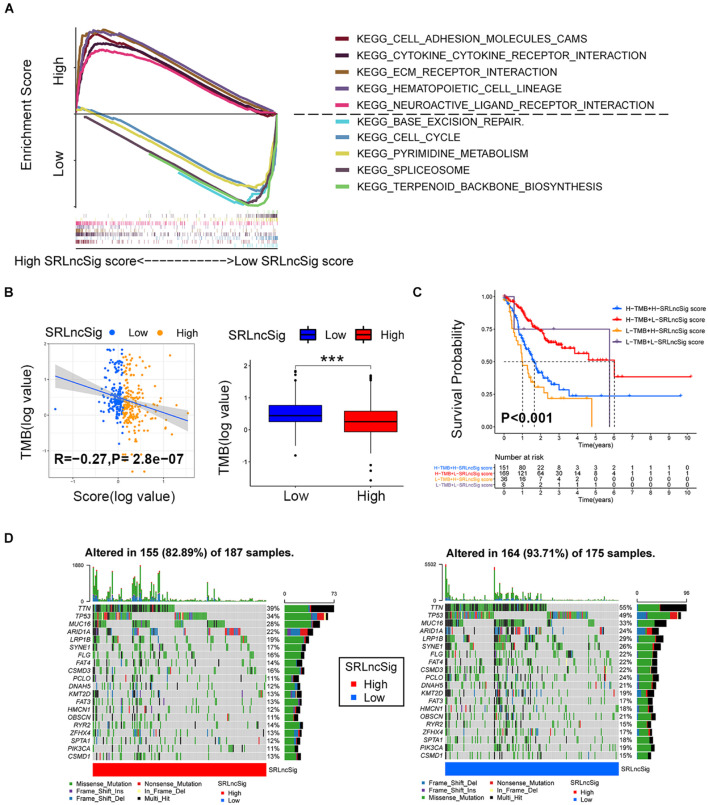
Enrichment analysis and the interrelations between the SRLncSig score and SNVs. **(A)** Top five enriched pathways enriched for high and low risk score, respectively. **(B)** The negative correlation between SRLncSig scores and tumor mutation burden (TMB) in the TCGA cohort was shown by scatter plots (Spearman text, *p* < 0.001). TMB variations between the high and low SRLncSig score groups (Wilcoxon test, ***p < 0.001). **(C)** The combination of TMB and SRLncSig score helped predict the overall survival. **(D)** The SNVs spectrum was constructed based on different SRLncSig score clusters, blue for low and red for high.

Based on the cutoff value of risk score, the TCGA cohort was divided into low- and high-risk groups to conduct GSEA analysis. The results revealed that the KEGG_CELL_ADHE SION_MOLECULES_CAMS, KEGG_CYTOKINE_CYTOK INE_RECEPTOR_INTERACTION, KEGG_ECM_RECEPTOR_INTERACTION, KEGG_HEMATOPOIETIC_CELL_LINEAGE, and KEGG_NEUROACTIVE_LIGAND_RECEPTOR_INTERA CTION were the top five most enriched pathways in the high SRLncSig score group, while the KEGG_BASE_EXCISION_REPAIR, KEGG_CELL_CYCLE, KEGG_PYRIMI DINE_METABOLISM, KEGG_SPLICEOSOME, and KEGG_TERPENOID_BACKBONE_BIOSYNTHESIS pathways were most enriched in the low SRLncSig score group (Figure 6A). Numerous studies supported the hypothesis that accumulating somatic mutation variants triggered the immune system to produce anti-cancer cells. Increased TMB was also identified as an indicator of improved response to PD-1 blockade and prolonged progression-free survival. Considering that the DNA repair concerning pathways were highly enriched, we investigated the intrinsic correlation between the TMB and SRLncSig scores to elucidate the genetic imprints of each subgroup. Based on the SNV data derived from TCGA-STAD, the TMB values for each sample were calculated. Then, Spearman correlation analysis also confirmed that the SRLncSig score was negatively correlated with the TMB values (Figure 6B). Furthermore, TMB values of the high and low SRLncSig score clusters were compared. The results indicated that patients in the low SRLncSig score subgroup showed a significantly higher TMB than patients in the low SRLncSig score subgroup (Figure 6B). The combination of TMB and SRLncSig score helped predict overall survival based on log rank test shown by Kaplan-Meier curves (Figure 6C). All of these results suggest that the SRLncSig might be an important predictive factor to indicate the immunotherapy response. To evaluate the potential driver mutations of GC, the mutated genes were listed using maftools. The result suggested that most of the mutated genes were higher in the low SRLncSig score group (93.71%) compared with the high score group (82.89%) (Figure 6D).

### Explorations of Clinical Applications for SRLncSig

**FIGURE 7:**
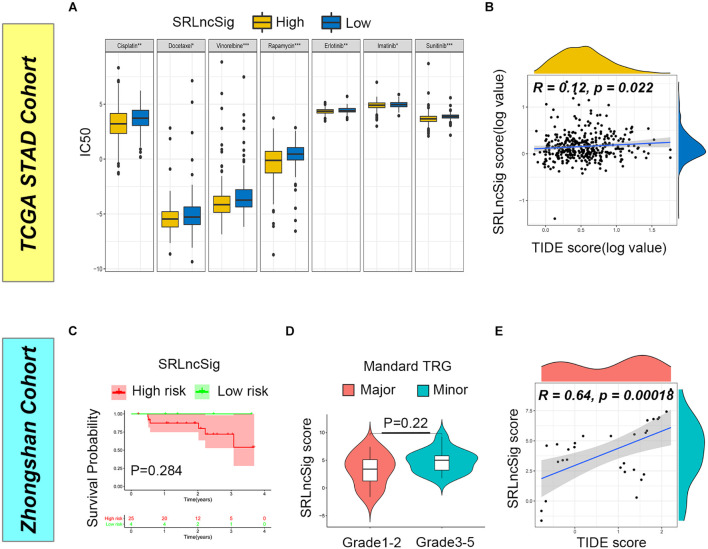
Predictive value of SRLncSig for therapeutic response. **(A)** Comparisons of IC50 for chemotherapeutics and targeted therapy drugs between two risk stratifications revealed that the high-risk group was more likely to benefit from the treatments (Kruskal-Wallis test, *p < 0.05; **p < 0.01; ***p < 0.001). **(B)** Positive correlation between SRLncSig and TIDE scores in the TCGA cohort was shown by scatter plots and lower SRLncSig score may be more likely to benefit from the immunotherapy (Spearman text, *p* < 0.05). **(C)** Kaplan-Meier estimates of overall survival of patients assigned as low- or high-risk group based on the cutoff score value. **(D)** The major response group also presented with a tendency of decreased score value. **(E)** Positive correlation between SRLncSig and TIDE score in the Zhongshan cohort was also observed (Spearman text, *p* < 0.001).

Firstly, we examined the efficacy of SRLncSig score for chemotherapy response prediction, and IC50s for each sample were calculated. Based on the pRRophetic algorithm and the drugs “pRRophetic” package, we analyzed seven chemotherapy (cisplatin, docetaxel, vinorelbine, rapamycin, erlotinib, imatinib, and sunitinib) and targeted therapy drugs that were used in GC treatment to predict the IC50 for each sample in two risk stratifications. The results revealed that the IC50 values for all drugs were significantly lower in the high SRLncSig score group (Figure 7A). The results could be partially explained by GSEA analysis, which revealed that a drug-resistant pathway like KEGG_BASE_EXCISION_REPAIR was highly enriched in the low SRLncSig score group. Those results confirmed that the SRLncSig score classification system might be a good predictor for chemotherapy response. Besides, we investigated the predictive value of SRLncSig score for immunotherapy response. IPS and TIDE score were two algorithms developed for immunotherapy response prediction. IPS was only available for the TCGA cohort and the TIDE score was acquired based on online calculation results. The IPS value for CTLA4 therapy response was higher in the low SRLncSig score group (Supplementary Figure 4) and the TIDE score was also positively correlated with SRLncSig score (Figure 7B). The results indicated that SRLncSig might also act as a predictor for immunotherapy.

To validate the results, the Zhongshan cohort including 29 patients receiving neoadjuvant chemotherapy were enrolled for analysis. Based on the cutoff value acquired from the TCGA cohort, 29 cases were divided into the low SRLncSig score group (*n* = 4) and the high SRLncSig score group (*n* = 25). Although no statistical difference was observed (*p* = 0.284), the low score group presented with tendency of better survival (Figure 7C). Since all the patients of the Zhongshan cohort had accepted neoadjuvant chemotherapy, we compared the SRLncSig score between the major response and minor response patients. The results indicated a decreased tendency of SRLncSig score for the major response patients (Figure 7D). We believed that the reason why no statistical significance is observed could be attributed to the relatively small number of samples. As for immunotherapy response prediction, a positive correlation was also observed between TIDE value and SRLncSig score (*R* = 0.64, *p* < 0.001) (Figure 7E). The results were highly consistent with our previous analysis.

**FIGURE 8:**
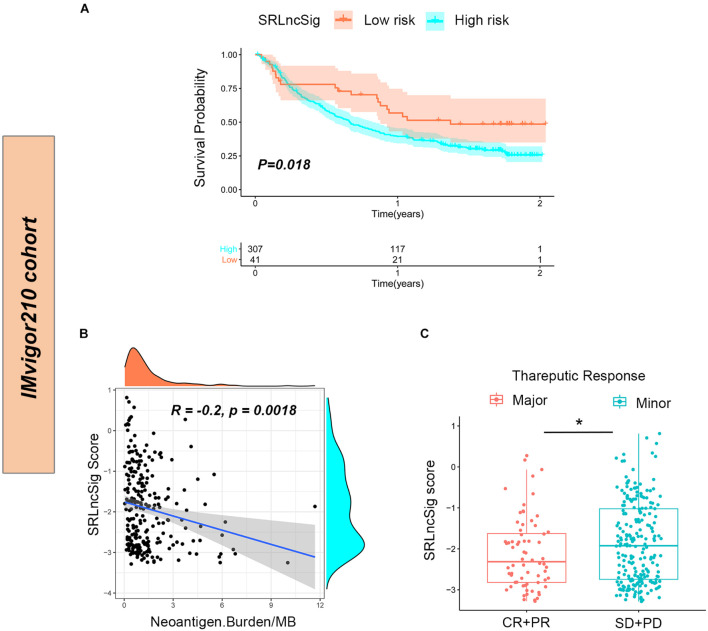
Predictive value of SRLncSig for immunotherapy response in the IMvigor210 cohort. **(A)** Kaplan-Meier estimates of overall survival of patients assigned as low- or high-risk group indicated that the low-risk group presented with better survival. **(B)** Negative correlation between SRLncSig score and neoantigen counts (Burden/MB) in the IMvigor210 cohort was shown by scatter plots and lower SRLncSig score may harbor higher level of neoantigens (Spearman text). **(C)** Comparison between the major and minor response groups indicated that a lower SRLncSig score might predict better immunotherapy response (Kruskal-Wallis test, *p < 0.05).

To further confirm that the SRLncSig score might serve as a predictor for immunotherapy response, the IMvigor210 cohort including 348 urothelial carcinoma patients who received immunotherapy were enrolled for analysis. Consistent with the results of the TCGA cohort, the low-risk group presented with better survival (*p* = 0.018) (Figure 8A). The counts of neoantigens were negatively correlated with SRLncSig score, suggesting that a higher level of immune events might exist in the cases with lower SRLncSig score (*R* = −0.2, *p* = 0.0018) (Figure 8B). The therapeutics results of the cohorts were available. The patients diagnosed as complete response (CR) and partial response (PR) were classified into the major response group while patients diagnosed as stable disease (SD) and progression response (PR) were classified into the minor response group. The SRLncSig score was also compared between major and minor response groups and lower SRLncSig score was observed in the major response group. The results validate the conclusion that SRLncSig might serve as an indicator for immunotherapy (Figure 8C).

## Discussion

After reviewing the literatures, we found that this was the first time that a SRLncSig with the capability of predicting immunotherapy response is reported in GCs. In this study, we aimed to develop a SRLncSig with guiding significance for immunotherapy therapy. The analysis was initiated by collecting SRlncRNAs in the TCGA STAD cohort based on the correlation analysis between SRGs and lncRNAs. The SRlncRNAs were further processed by univariate Cox regression analysis to find out the lncRNAs with prognosis-predictive value. After that, the collected lncRNAs were acquired to establish the SRLncSig risk score model with Lasso regression analyses. Since stemness was acknowledged as an important cause of chemotherapy resistance (Grandits and Wieser, 2021; Liu et al., 2021; Marimuthu et al., 2021; Narusaka et al., 2021; Oliveira et al., 2021; Panicker et al., 2021), the SRLncSig was firstly tested for its predictive value for chemotherapy response. Higher SRLncSig score was revealed with lower IC50, suggesting a superior chemotherapy response for the high-risk group. However, the results were inconsistent with the Zhongshan cohort. Considering that only 29 cases were included in the Zhongshan cohort, it was not enough to make people believe that the signature could serve as an effective predictor of chemotherapy. Notably, stemness has also been reported to be associated with immunotherapy in recent published literatures (Vodnala et al., 2019; Hadadi et al., 2020; Ishii et al., 2021; Tomei et al., 2021; Wang et al., 2021). It prompted us to explore the relevance between the SRLncSig and immunotherapy. We further examined the predictive efficacy of immunotherapy in the TCGA cohort. IPS analysis and TIDE analysis were two methods most frequently applied in bioinformatic studies (Wu et al., 2021; Xu R. et al., 2021; Xu Y. et al., 2021; Zhao et al., 2021; Zuo et al., 2021). The IPS analysis results suggested that the SRLncSig might present a better performance in predicting CTLA4 blockade therapy. A positive correlation between SRLncSig and TIDE scores in TCGA cohort was shown by scatter plots and a lower SRLncSig score may be more likely to benefit from the immunotherapy. The conclusions of the two algorithms are relatively consistent. Afterward, the Zhongshan cohort was examined again to calculate the TIDE scores. Though the SRLncSig did not present with statistical significance for prognosis and chemotherapy response predictions, a positive correlation between SRLncSig score and TIDE score was consistently observed. The above results strongly suggested that SRLncSig might serve as an effective predictor for immunotherapy. To finally prove the hypothesis, the IMvigor210 cohort including 348 urothelial carcinoma patients who received immunotherapy were applied to test the immunotherapy predictive value of SRLncSig. Similar analyses across tumor species have been applied in previous studies (Zhang B. et al., 2020; Chen H. et al., 2021; Chong et al., 2021). The results were also highly matched with the previous results.

With the popularity and development of transcriptome sequencing technology in recent years, GC-related studies focusing on constructing signatures with both coding genes and non-coding RNAs to assist with clinical decision-making are increasing (Chen et al., 2020; Chen Y. et al., 2021). Though cancer stemness, lncRNAs, and tumor immunity have all emerged as important factors of cancer in recent years, their covariation across cancers has not been systematically elucidated. As one of the most significant characteristics, CSCs have attracted increasing attention for their self-renewal and multipotent properties, as well as their proliferative potential, which gives certain cellular subpopulations the ability to initiate, develop, and progress to cancer (Huang et al., 2020). Previous studies have disproved the connection between lncRNAs and cancer stemness. Zhu et al. (2018) discovered that a Gata6 long non-coding RNA (lncGata6) is highly expressed in intestinal stem cells. lncGata6 helped maintain stemness of intestinal stem cells and promoted intestinal tumorigenesis (Zhu et al., 2018). A novel lncRNA named lnc030 was also revealed to maintain breast cancer stem cell stemness by stabilizing SQLE mRNA. lnc030 could also cooperate with poly(rC) binding protein 2 (PCBP2) to stabilize squalene epoxidase (SQLE) mRNA, resulting in an increase of cholesterol synthesis (Qin et al., 2021). Some of the lncRNAs included in our signature have been reported as contributors to the stemness of tumors. ADAMTS9-AS2 was reported to serve the function of controlling the chondrogenic differentiation by acting as a competing endogenous RNA (ceRNA) in human mesenchymal stem cells (hMSCs; Huang et al., 2019). RNF144A-AS1 could act as a regulator of mesenchymal stem cell (MSC) chondrogenesis *via* suppression of the interferon type II signaling pathway (Huynh et al., 2020). The above findings suggested that the lncRNAs included in SRLncSig were worthy of further studies.

It is understandable that the stemness-related signature served as a predictor for cytotoxic chemotherapeutics. However, it remained obscure why the signature presented with similar utility for targeted therapies. After reviewing the literatures, we found that targeted drugs may block target proteins that were crucial for the development of stemness properties. For example, hypoxia-inducing factors such as HIFs have been reported to enhance stemness properties in multiple malignancies. Drugs targeting these factors, such as “TH-302,” have been revealed to overcome chemotherapy resistance caused by stemness-related pathways (Mimeault and Batra, 2013). As contributors to stemness, YAP and TAZ were frequently observed in various cancers and were associated with chemotherapeutic resistance. Dasatinib may inhibit the nuclear localization and target gene expression of YAP and TAZ and thus reverse chemotherapy resistance (Oku et al., 2015). However, direct evidence of our hypothesis is still limited.

Numerous studies have been reported to investigate whether tumor immune microenvironment could be affected by stemness features. Mao et al. (2021) developed a mRNA expression-based stemness index system based on weighted gene correlation network analysis to reveal that the stemness index indicated a distinct immune spectrum. Alex et al. used gene expression-based metrics to evaluate the association of stemness with immune cell infiltration and genomic, transcriptomic, and clinical parameters across 21 solid cancers. The authors found pervasive negative associations between cancer stemness and anticancer immunity (Miranda et al., 2019). Ma et al. (2018) also proposed the opinion that glioma stem cells (GSCs) and other non-tumor cells existing in the glioma microenvironment could serve as critical regulators of the immune landscape. The accumulation of stem cells is highly correlated with the immunosuppressive microenvironment in glioma (Ma et al., 2018). Considering that distinct tumor immunity was observed, it is reasonable to hypothesize that immunotherapy efficacy could be predicted by SRLncSig. The results of our analysis did partially confirm the hypothesis.

In recent years, studies committing to constructing stemness-related models for clinical decision guidance have emerged. Zhang C. et al. (2020) built a 13-gene-based prostate cancer stemness model that had high predictive significance for progression-free survival (PFS). The model was also revealed to be closely linked to immune microenvironment changes (Zhang C. et al., 2020). Wang et al. (2021) established a novel stemness-based classification with appealing implications in discriminating the prognosis and immunotherapy and temozolomide responses of 906 glioblastoma patients. Interestingly, both of the studies concluded that stemness features might serve as a predictor of immunotherapy. The results are highly consistent with our studies.

As for the selection of appropriate patients for ICB therapy, the criteria are still controversial. Of all the reported standards, the Combined Positive Score (CPS) of PD-L1 attracts the most attention. The CPS focuses not only on the percentage of PD-L1-positive cells but also sheds light on the cell types (Rosenbaum and Gonzalez, 2021). However, the CPS remains as the standard of manual reading. The efficacy and accuracy of the CPS has actually not been well elucidated (Wainberg et al., 2021). Recently, an inflammatory gene signature was tested for its efficacy to predict the treatment response of ICB treatments for GCs (Lei et al., 2021). Considering that the criteria were established based on objective indexes, the detection stability may be superior compared with CPS. Therefore, the signature we established was worthy of further study for its predictive efficacy. Some limitations in this study inevitably exist. Although a high predictive efficacy was observed in TCGA STAD datasets, we failed to obtain an immunotherapy-concerning GC cohort to validate the practicability of this model. In addition, all predictive results were derived from bioinformatic methods. A real-world analysis is urgently needed.

## Conclusion

We firstly collected SRlncRNAs based on co-expression analysis between SRGs and lncRNAs in the TCGA cohort. After that, we established a SRLncSig for the prediction of GC patient survival and immunotherapy response. This study not only provided a novel signature with prognostic predictive value in GC patients but also established a model to predict GC patients’ sensitivity to immunotherapy. A lower SRLncSig score was revealed as an indicator for immune checkpoint applications. The efficacy of SRLncSig was further validated by the Zhongshan cohort and the IMvigor210 cohort.

## Data Availability Statement

The original contributions presented in the study are included in the article/Supplementary Material, further inquiries can be directed to the corresponding authors.

## Ethics Statement

The studies involving human participants were reviewed and approved by the Ethics Committee of Zhongshan Hospital Affiliated to Fudan University. The patients/participants provided their written informed consent to participate in this study.

## Author Contributions

QJ and LC performed the data analysis and wrote the manuscript. ZT, FL, and YS designed the study. HC contributed to reviewing and editing the article. FL and YS provided funding acquisition. All authors read and approved the final submitted manuscript.

## Conflict of Interest

The authors declare that the research was conducted in the absence of any commercial or financial relationships that could be construed as a potential conflict of interest.

## Publisher’s Note

All claims expressed in this article are solely those of the authors and do not necessarily represent those of their affiliated organizations, or those of the publisher, the editors and the reviewers. Any product that may be evaluated in this article, or claim that may be made by its manufacturer, is not guaranteed or endorsed by the publisher.

## Footnotes

1
https://portal.gdc.cancer.gov/

2
http://asia.ensembl.org

3
https://tcia.at/home

4
http://tide.dfci.harvard.edu

## Supplementary Material

Supplementary Figure 1 Univariate Cox regression analysis revealed 66 SRLncRNAs with prognosis-predictive value in training cohort.

Supplementary Figure 2 Correlation analysis of 66 SRLncRNAs (Spearman method, ^∗^*p* < 0.05, red plot for positive correlations; blue plot for negative correlations).

Supplementary Figure 3 23 out of 66 SRLncRNAs derived from prior Cox regression analysis are collected to develop the SRLncSig by Lasso regression algorithms. Different colors represented different lncRNAs.

Supplementary Figure 4 IPS analysis revealed that the SRLncSig may present a better performance in predicting CTLA4 blockade therapy compared to PD1 blockade therapy (Mann-Whitney *U* test, ^∗∗^*p* < 0.01; ns for no statistical difference).

Supplementary Table 1 The clinical baseline of training and texting cohorts.
