# Supplementary material for: Integrated Analysis of Stemness-Related LncRNAs Helps Predict the Immunotherapy Responsiveness of Gastric Cancer Patients
Source: Front Cell Dev Biol. 2021 Sep 13;9:739509. doi: 10.3389/fcell.2021.739509 (PMC8473797; doi:10.3389/fcell.2021.739509)
Supplement: Supplementary Figure 1 — Univariate Cox regression analysis revealed 66 SRLncRNAs with prognosis-predictive value in training cohort. [file Data_Sheet_1.docx]

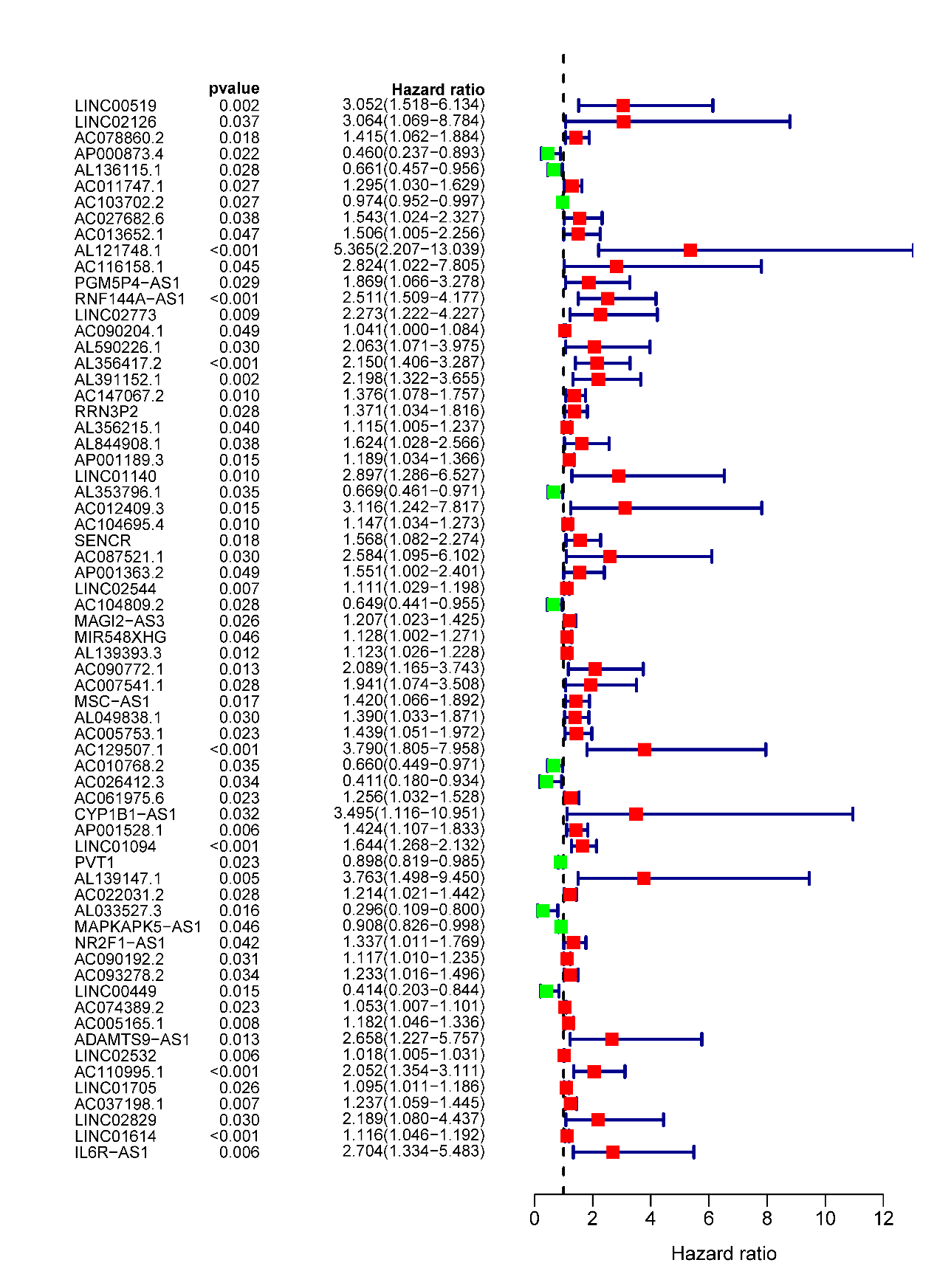
 sFigure 1. Univariate Cox regression analysis revealed 66 SRLncRNAs with prognosis-predictive value in training cohort.


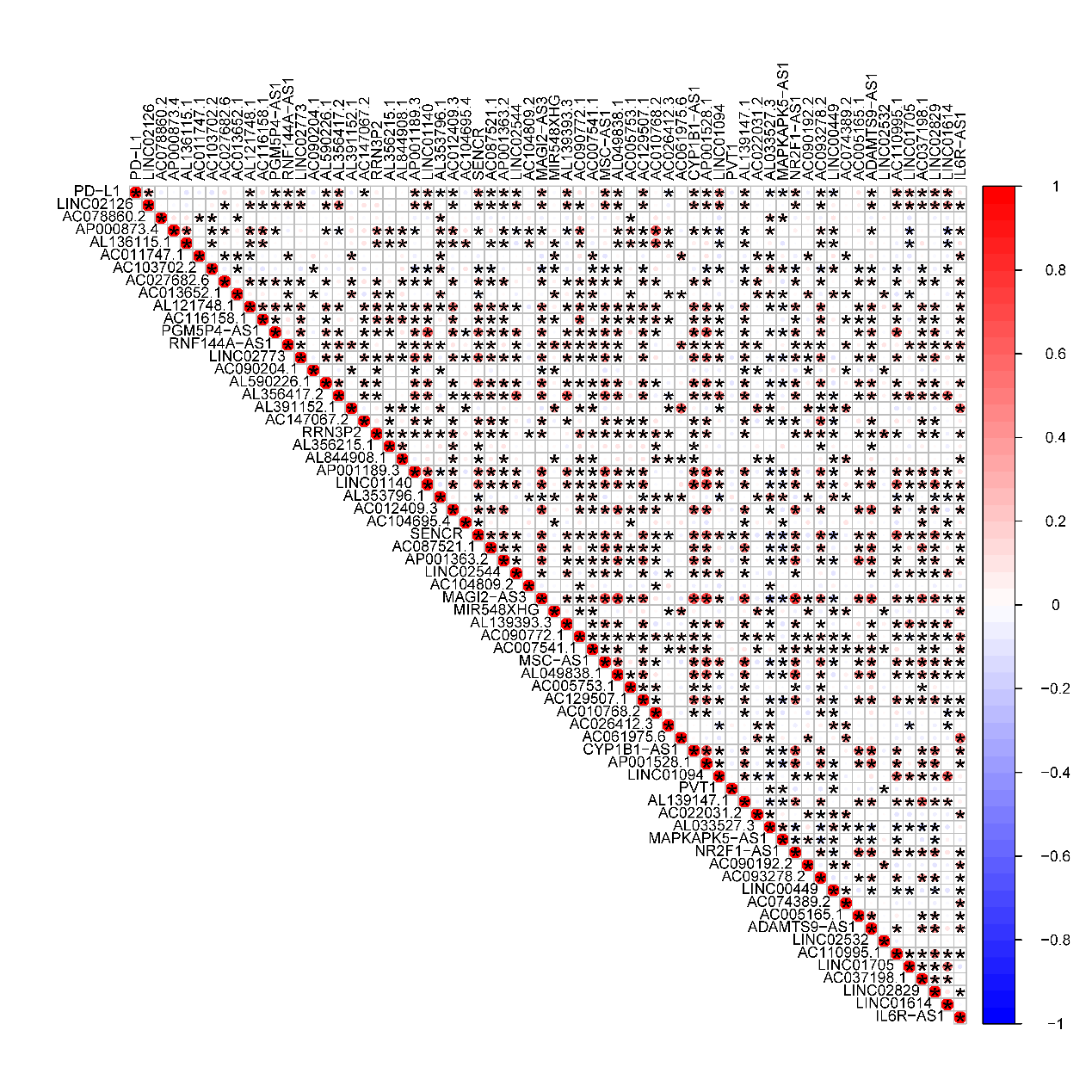
sFigure 2. Correlation analysis of 66 SRLncRNAs. (Spearman method. *p < 0.05, red plot for positive correlations; blue plot for negative correlations)


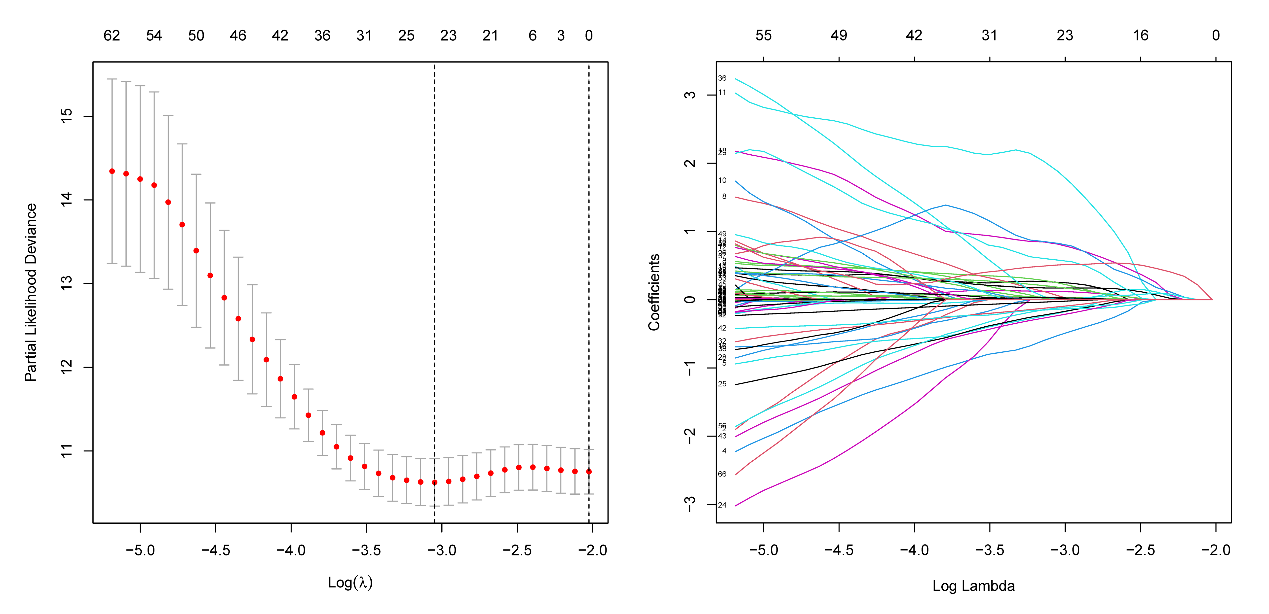


sFigure 3. 23 out of 66 SRLncRNAs derived from prior Cox regression analysis are collected to develop the SRLncSig by Lasso regression algorithms. (Different colors represented different lncRNAs)


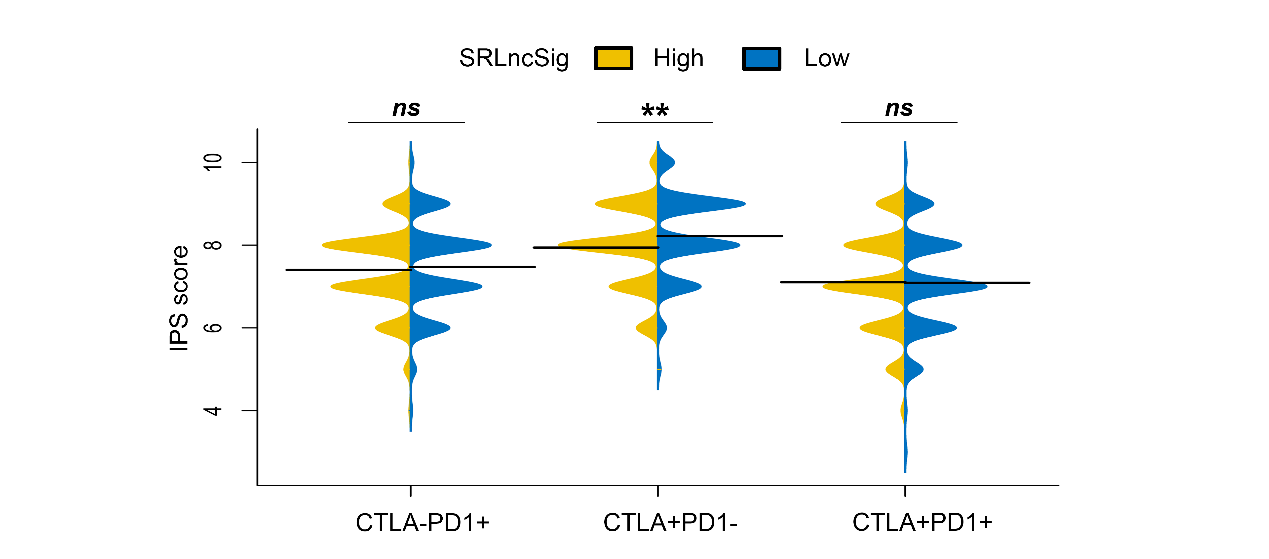


sFigure 4. IPS analysis revealed that the SRLncSig may present a better performance in predicting CTLA4 blockade therapy compared to PD1 blockade therapy. (Mann-Whitney U test. **p < 0.01; ns for no statistical difference).

| sTable 1 The clinical baseline of training and texting cohorts. | | | | | |
| --- | --- | --- | --- | --- | --- |
| Covariates |  | Total | Test | Train | P-value |
| Age | <=65 | 163(43.94%) | 81(44.02%) | 82(43.85%) | 1 |
|  | >65 | 205(55.26%) | 102(55.43%) | 103(55.08%) |  |
|  | unknow | 3(0.81%) | 1(0.54%) | 2(1.07%) |  |
| Gender | FEMALE | 133(35.85%) | 64(34.78%) | 69(36.9%) | 0.7515 |
|  | MALE | 238(64.15%) | 120(65.22%) | 118(63.1%) |  |
| Grade | G1-2 | 144(38.81%) | 67(36.41%) | 77(41.18%) | 0.4775 |
|  | G3 | 218(58.76%) | 111(60.33%) | 107(57.22%) |  |
|  | unknow | 9(2.43%) | 6(3.26%) | 3(1.6%) |  |
| Stage | Stage I-II | 161(43.4%) | 72(39.13%) | 89(47.59%) | 0.0688 |
|  | Stage III-IV | 187(50.4%) | 103(55.98%) | 84(44.92%) |  |
|  | unknow | 23(6.2%) | 9(4.89%) | 14(7.49%) |  |
| T | T1-2 | 96(25.88%) | 42(22.83%) | 54(28.88%) | 0.2245 |
|  | T3-4 | 267(71.97%) | 138(75%) | 129(68.98%) |  |
|  | unknow | 8(2.16%) | 4(2.17%) | 4(2.14%) |  |
| M | M0 | 328(88.41%) | 159(86.41%) | 169(90.37%) | 0.6045 |
|  | M1 | 25(6.74%) | 14(7.61%) | 11(5.88%) |  |
|  | unknow | 18(4.85%) | 11(5.98%) | 7(3.74%) |  |
| N | N0 | 108(29.11%) | 49(26.63%) | 59(31.55%) | 0.2824 |
|  | N1-3 | 245(66.04%) | 128(69.57%) | 117(62.57%) |  |
|  | unknow | 18(4.85%) | 7(3.8%) | 11(5.88%) |  |
